# Physical Activity of Working-Age People in View of Their Income Status

**DOI:** 10.1155/2018/8298527

**Published:** 2018-11-01

**Authors:** Daniel Puciato, Michał Rozpara, Władysław Mynarski, Piotr Oleśniewicz, Julita Markiewicz-Patkowska, Małgorzata Dębska

**Affiliations:** ^1^Faculty of Finance and Management, WSB University in Wrocław, Ul. Fabryczna 29-31, 53-609 Wrocław, Poland; ^2^Faculty of Physical Education, The Jerzy Kukuczka Academy of Physical Education in Katowice, Ul. Mikołowska 72, 40-065 Katowice, Poland; ^3^Faculty of Physical Education, University School of Physical Education in Wrocław, 35 Ignacego Jana Paderewskiego Street, 51-612 Wrocław, Poland

## Abstract

**Objective:**

The study examines relationships between physical activity levels and income status of working-age city residents.

**Methods:**

The study was carried out in the years 2014 and 2015 in Wrocław, Poland. The study sample comprised 4332 participants (2276 women; 2056 men) aged 18 to 64 years. Respondents' habitual physical activity levels were measured with the International Physical Activity Questionnaire Short Form (IPAQ-SF), while their income status was assessed with author's own Socio-Economic Status of Working-Age People Questionnaire (S-ESQ).

**Results:**

The results revealed positive correlations between the level of physical activity and income status of male and female working-age residents of Wrocław. The highest physical activity levels were noted among respondents with a steady income, as well as among respondents with the highest income and savings and with no debts. The odds for respondents' above average physical activity levels were the greatest in women with the highest income and with savings and in debt-free men and women.

**Conclusion:**

Effective actions should be developed aimed at improvement of physical activity levels of people in an adverse financial situation.

## 1. Introduction

Undertaking properly adjusted physical activity by working-age populations is highly significant for their health status in its physical, psychical, and social domains. Significant positive correlations were empirically proven between physical activity levels and the function of locomotive [1, 2], circulatory [3, 4], respiratory [5], digestive [6], immune [7], and nervous [8] systems. Researchers also confirmed the positive impact of physical activity on anxiety and depression levels [9], cognition [10], optimism [11], and quality of life [12–15]. Physical exercises of appropriate frequency, volume, and intensity play a crucial role in disease prevention as well as in rehabilitation allowing full recovery and return to professional life after diseases, traumas, or exhaustion [16, 17].

Following the ecological model proposed by Sallis et al. [18] an individual's physical activity is determined by different sets of variables: intrapersonal, interpersonal, environmental, regional, national, and global. The first set includes, apart from biological determinants (genes; health status) and psychological determinants (motivation, cognition, values, and emotions), also socio-economic factors, i.e., one's age, sex, education, occupation, and income status.

The present study attempts to identify relationships between physical activity levels of working-age individuals and their income status. Results of earlier studies examining these relationships have been rather inconclusive. Chung et al. [19] analyzed the levels of physical activity of adult Americans in relation to their wealth and occupation status in the years 1996-2002. The study showed that individuals with the annual household income above 125 thousand dollars featured higher physical activity levels than people with lower household income levels. Also Kim and So [20] in their study of a Korean population noted a strong relationship between higher physical activity levels among people with a higher income. A study of Warsaw residents by Biernat [21] revealed that the odds of meeting WHO recommended levels of physical activity increased with the residents' higher income status. The odds of fulfillment the WHO standards among people with the highest income were almost twice as high as among people with the lowest income.

Studies of physical workers from Brazil [22] and China [23] revealed the highest levels of physical activity among individuals with the highest and the lowest income. Kaewthummanukul and Brown [24] and Van Stralen et al. [25] noted that economic status was not significantly correlated with undertaken physical activities, whereas Sallis et al. [26] found negative correlations between income status and physical activity levels among adults.

Researchers so far have investigated study participants' financial situation only in terms of household income or income status of particular household members; however, no other significant variables have been considered. The aim of the present study was to identify the strength and direction of relationships between physical activity levels and such determinants of financial situation as having steady income, per capita income, savings, and indebtedness in a working-age population from Wrocław, Poland.

## 2. Methods

The study was carried out between 2014 and 2015 in the city of Wrocław (pop. 640 000) in Poland. The research project had been given a positive opinion by the Commission of Bioethics at the University of Physical Education in Wrocław. The research sample comprised 4,332 people (2,276 women; 2,056 men) aged 18-64 years, i.e., about 1% of the Wrocław working-age population. The respondents were divided into the following age ranges: 18-24 years (14%), 25-34 years (26%), 35-44 years (20%), 45-54 years (17%), and 55-64 years (23%). Among the respondents 31% were white-collar workers, 26% manual workers, 14% self-employed and students, 9% unemployed, and 6% homemakers. Almost 81% of the Wrocław residents under study had a steady income. 45% had the average monthly income per capita from USD 271 to 542; 28% below USD 271; and 27% more than USD 542. About 46% had money savings, and nearly 54% had no spare funds whatsoever. 49% of respondents were in debt, and 51% had no financial liabilities. The differences in financial status between the male and female respondents were statistically significant (p < 0.001) (Table 1); thus the analysis of relationships between physical activity levels and income status was carried out for men and women separately.

The study used the auditorium survey methodology. The respondents' physical activity and financial situation were assessed with the International Physical Activity Questionnaire Short Form (IPAQ-SF) in the Polish language version [27] and author's own Socio-Economic Status of Working Age People Questionnaire (S-ESQ).

The data from IPAQ-SF was used to determine respondents' energy expenditure of physical activity (EEPA) (quantitative index) and physical activity level (PAL) (qualitative index). The EEPA expressed in MET-min/week was calculated as a total of physical activities at three intensity levels performed by respondents on a weekly basis [27]. The PAL was calculated as a scaled up (adapted) EEPA index. The reference category, in the groups of men and women separately, was the EEPA median value (Me EEPA). The two PAL categories were average PAL (EEPA ≤ Me EEPA) and above average PAL (EEPA > Me EEPA).

The S-ESQ was used to determine four independent variables of the income status of Wrocław residents: having a steady income (YES, NO), per capita income in a household (< USD 271, USD 271-542, USD > 543), savings (YES, NO), and indebtedness (NO, YES).

For each variable, the size (n) and ratios (%) for the gender groups and the whole study group were estimated. As for the EEPA (dependent variable) arithmetic means and medians (Me) were calculated for the total group and groups according to particular variables of the financial situation, for men and women separately. The differences between the male and female Wrocław residents were checked with Pearson's chi-squared test (*χ*^2^). Differences in the average physical activity levels between respondents with different income status were checked with the use of the Mann-Whitney *U* test (Z), Kruskal-Wallis ANOVA test (H), and logistic regression analysis, separately for male and female residents. The level of statistical significance was set* ex ante* at *α* < 0.05. All statistical calculations were made with the use of IBM SPSS Statistics 20.

## 3. Results

**Figure 1:**
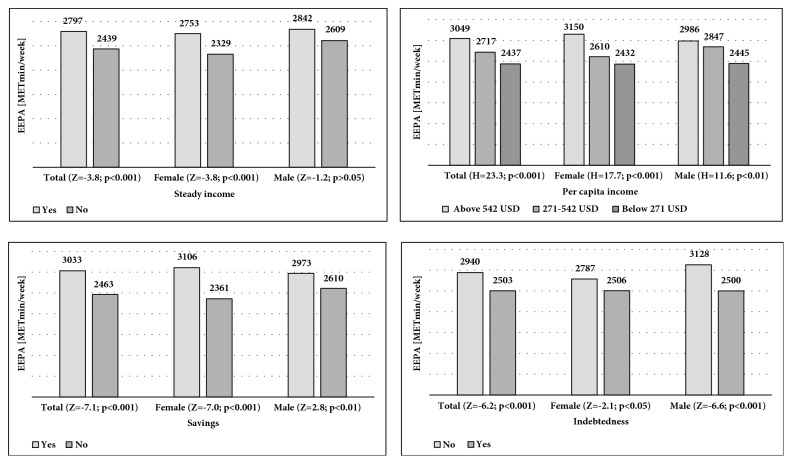
Differences in physical activity levels of respondents grouped according to variables of their income status.

The analysis of mean EEPA results in the groups of male and female respondents with regard to having a steady income revealed significant differences in the group of women (Z = -3.8, p < 0.001). The mean EEPA among women with a steady income was 2753 MET-min/week, and among women without a steady income 2329 MET-min/week. The highest levels of physical activity were noted in women (3150 MET-min/week) and men (2986 MET-min/week) from Wrocław with incomes higher than USD 542, and the lowest in women (2432 MET-min/week) and men (2445 MET-min/week) with incomes below USD 271. The Kruskal-Wallis test results (H = 17.7, p < 0.001 in women; and H = 11.6, p < 0.01 in men) showed that mean physical activity levels in particular income groups varied significantly. The highest EEPA levels were found in women (3106 MET-min/week) and men (2973 MET-min/week) who had money savings. Their level of physical activity was significantly higher (p < 0.001 in women; p < 0.01 in men) than in respondents without savings (2361 MET-min/week in women; 2610 MET-min/week in men). The respondents' physical activity levels also differed in regard to their indebtedness (Z = -2.1, p < 0.05 for women; Z = -6.6, p < 0.001 for men). The mean EEPA (2787 MET-min/week in women; 3128 MET-min/week in men) in debt-free respondents was higher than in respondents with debt, i.e., 2506 MET-min/week and 2500 MET-min/week, respectively (Figure 1).

Tables 2 and 3 show binomial regression models illustrating relationships between physical activity levels (PAL) (dependent variable) with income status indices of working-age Wrocław residents (independent variables). The reference category for the PAL index was its average level, i.e., below or equal to 2232 MET-min/week in women and below or equal to 2445 MET-min/week in men.

Among the female residents statistically significant relationships were found between the physical activity level and per capita income (p < 0.001), savings (p < 0.01), and indebtedness (p < 0.05). The odds ratio of the women's PAL being above average was 80% greater in the ones with the highest income than in those with the lowest income per capita. Women from Wrocław with money savings had 35% greater odds of above average PAL than women without any money savings. Also the odds of above average PAL in debt-free female residents from Wrocław were 24% higher than in women with debt (Table 2). In men, statistically significant correlations between the physical activity level and income status variables were only found in men with debt (p < 0.001). The odds of an above average PAL were 60% greater in debt-free men than in men with financial liabilities (Table 3).

## 4. Discussion

The first variable of income status, which had not been considered in earlier research, was having a steady income. Respondents with steady incomes who had higher levels of physical activity were fully employed or self-employed. The relatively short time after Poland's transformation from a centrally planned to free market economy has made only a small part of Polish society depend on a steady income from the held capital, rather than not from work. People who work full-time are better organized and generally more physically active than the unemployed [28]. They often undertake specific physical activity by commuting and performing their professional tasks. The significance of physical exercise during professional work and commuting as part of the overall physical activity of adults was proven before [29]. Researchers also noted higher physical activity levels among white and blue collar workers than among the unemployed [30].

The physical activity levels of the Wrocław residents under study were significantly different with regard to respondents' income per capita. An increase in PAL with a higher income per capita was noted by Biernat [21], Choi et al. [31], Kim and So [20], and Kari et al. [32]. Interestingly, there was an increase in conditional probability of undertaking above average physical activity in individuals with the highest income per capita. A significant increase in the physical activity level after crossing a certain income threshold was also found by Chung et al. [19]. The possibility of undertaking more physical activity by individuals with the highest incomes can be interpreted in a twofold way. On the one hand, it can be explained by the substantial contribution of physical exercises related to the performance of professional chores and of commuting to one's total physical effort. High income is often derivative of the time, complexity, and intensity of one's occupation. On the other hand, the impact of high income on pursuing leisure physical activity is also significant. This is related to the so-called backward-bending labor supply curve which demonstrates that higher wages actually entice people to work less and consume more leisure [33]. Some individuals with high incomes experience the need to have more leisure. With a higher income employees can reduce their work time, e.g., by cutting extra hours or working part-time, affording free weekends, going on vacation more than once a year, or even considering an earlier retirement. On the other hand, individuals with a low income sometimes use part of their free time for extra-paid work. This may allow them to fully meet all household needs but also deplete their energy resources that can be otherwise utilized for undertaking free-time physical activity. Thanks to a high income one is able to purchase various goods and services directly (e.g., gym subscriptions) or indirectly (e.g., transportation to and from physical exercise facilities). The above can be confirmed by research into quality of life showing that in 2015 the percentage of adult Poles spending their leisure time actively (walking and practicing outdoor leisure activities or sports) increased in particular income quintile groups [34]. Positive correlations between leisure physical activity and income status were also found by Jurakic et al. [35], Piko and Keresztes [36], and Stamm and Lamprecht [37].

Other indices allowing a complex assessment of respondents' economic situation are savings and indebtedness. Apart from current income (wages, social benefits, financial aid, rentals, etc.) household wealth is also determined by capital revenue, money savings, and accumulated material assets (e.g., real estate, works of art, jewelry, and consumer durables). The Wrocław residents with savings were more physically active than residents without savings. In 2015, 45% of Poles had money savings. The majority of them had savings amounting to one to three monthly salaries [38]. In view of the above it can be concluded that in the Polish economic reality having savings is still the preserve of a rather narrow group of affluent individuals. In fact, Poland constitutes a rather specific case. The national average propensity to save, i.e., savings rate, in Poland is both lower than in more economically advanced countries and in other Central and Eastern European countries at a similar economic level [38]. The other group of people with money savings are those whose income is not high, but who display such character traits as thriftiness, sense of control of one's own life, and foresightedness. These traits may also determine health-related behaviors including properly adjusted physical activity [39, 40].

The volume of physical activity undertaken by adult residents of Wrocław was also significantly affected by their indebtedness. Debt-free residents, compared with residents with debts, had also higher odds to reach an above average level of physical activity. Indebtedness is currently a serious problem in many Polish households: in 2015 about 32% of Poles were in debt. For most of them their debt exceeded the annual household income. The most frequent forms of debt include bank credits and loans, e.g., real estate mortgages, home renovation loans, or consumer durables loans [38]. The necessity to pay debts, regardless of debtors' financial status, always negatively affects the debtor's income status and fulfilment of needs. In the first place, potential savings are those for addressing further needs whose nonfulfilment is not health- or life-threatening, e.g., leisure needs, including physical activity needs [41]. Indebtedness also contributes negatively to one's functioning in social life and lowers one's quality of life, which is also significant for health-related behaviors, including undertaking physical activity, by working-age people [14, 15, 42, 43].

The present study has its strong and weak points. One of the first strong points is the much broader age range of the respondents (18-64 years) than in earlier studies. Only few studies had also focused on Poland or other countries of Central Europe. A novel contribution of the present study is also the focus on relationships of physical activity with such economic variables as steady income, savings, and indebtedness. The consideration of this complex set of variables permitted a comprehensive assessment of relationships between the physical activity and income status of working-age individuals that had not been attempted before in available literature. The study also makes use of novel methods of data analysis, especially in reference to the PAL index categories identified on the basis of descriptive statistics (intragroup differences between the physical activity levels of Wrocław residents).

A weak point of the present research is the confinement of the study area to a single city. Future studies should cover the entire area of Poland and other Central European countries. A certain shortcoming of the present study is the use of the short version of the IPAQ. Again future research should take advantage of measurements of physical activity in various areas of life, e.g., during leisure, work, commuting, or performing domestic chores, since the strength and directions of correlations with different socio-economic factors may vary [29]. A certain shortcoming of the present study was the analysis of the impact of particular socio-economic variables separately from one another. Future research should focus on correlations of the physical activity of working-age individuals with particular socio-economic factors as well as with the whole socio-economic situation of a household. Aggregate socio-economic indices were earlier considered by authors, but they were mostly used to examine the effects of socio-economic status on the somatic and motor development of young people [44–46]. Other indices of respondents' socio-economic status to be considered in the future should also encompass levels of steady income, amount of money savings, and different types of debts, e.g., short- or long-term.

## 5. Conclusion

The study results indicate positive relationships between physical activity levels and financial situation of a working-age population from Wrocław, Poland. The highest levels of physical activity were found among both male and female respondents who had the highest income, were debt-free, and had money savings. The study also showed that the odds for above average physical activity levels were the greatest in women with the highest income, who had money savings, and in debt-free men and women.

Since the study of physical activity of working-age people has significant and broad practical implications for public health, it is necessary to seek new and effective remedial programs regarding hypokinesia. The results of the study show that individuals who are prone to hypokinesia include people with low income, with no money savings, and with debt. Actions aimed at improving the level of physical activity should be initiated by these individuals themselves as well as by socio-economic entities (companies, associations, and foundations) and institutional bodies. Examples of such actions may include developing, financing, and implementing programs aimed at increasing physical activity levels; tax exemptions and advantages, e.g., lower taxes on sport and recreational goods and services; public awareness campaigns promoting physical activity; or preferential health insurance terms for physically active individuals. A high level of physical activity, and in consequence, better health status and quality of life can in turn greatly contribute to higher work efficacy of working-age individuals as well as to better economic output of business companies and national economies.

## Figures and Tables

**Table 1:** Income status of working-age residents from Wrocław.

Variables	Category	Total		Women		Men		*χ* ^2^	p
		n = 4332		n = 2276		n = 2056			
		n	%	n	%	n	%		
Steady income	Yes	3494	80.7	1765	77.5	1729	84.1	29.68	< 0.001
	No	838	19.3	511	22.5	327	15.9		

Per capita income	Below USD 271	1214	28.0	750	33.0	464	22.6	137.28	< 0.001
	USD 271-542	1957	45.2	1078	47.4	879	42.8		
	Above USD 542	1161	26.8	448	19.7	713	34.7		

Savings	Yes	2009	46.4	907	39.9	1102	53.6	82.11	< 0.001
	No	2323	53.6	1369	60.1	954	46.4		

Indebtedness	Yes	2107	48.6	1049	46.1	1058	51.5	12.47	< 0.001
	No	2225	51.4	1227	53.9	998	48.5		

Notes: *χ*^2^: chi-squared independence test; *p*: chi-squared independence test probability value.

**Table 2:** Relationships between physical activity level and income status variables in women.

Variables	Category	*β*	SE	Wald *χ*^2^	*p*-value	OR	CI	
							-95%	95%
	Intercept	-0.27	0.11	6.44	< 0.01			
Steady income^a^	Yes	0.09	0.10	0.80	≥ 0.05	1.10	0.89	1.35
Per capita income^b^	USD 271-542	-0.07	0.10	0.47	≥ 0.05	0.93	0.76	1.14
	above USD 542	0.59	0.18	10.58	<0.001	1.80	1.26	2.57
Savings^c^	Yes	0.30	0.10	9.87	< 0.01	1.35	1.12	1.63
Indebtedness^d^	No	0.22	0.09	6.34	< 0.05	1.24	1.05	1.47

Notes: the reference category for the dependent variable is average level of physical activity. ^a^The reference category for steady income is NO. ^b^The reference category for per capita income is below USD 271. ^c^The reference category for savings is NO. ^d^The reference category for indebtedness is YES. *β*: assessment value of model parameters, SE: asymptotic standard error *β*, Wald *χ*^2^: parameter significance, *p*: Wald *χ*^2^ probability value, OR: odds ratio, and CI: confidence interval.

**Table 3:** Relationships between physical activity level and income status variables in men.

Variables	Category	*β*	SE	Wald *χ*^2^	*p*-value	OR	CI	
							-95%	95%
	Intercept	-0.47	0.13	12.94	<0.001			
Steady income^a^	Yes	0.19	0.13	2.11	≥ 0.05	1.21	0.94	1.56
Per capita income^b^	USD 271-542	0.03	0.12	0.05	≥ 0.05	1.03	0.81	1.31
	Above USD 542	0.05	0.13	0.15	≥ 0.05	1.05	0.81	1.37
Savings^c^	Yes	0.03	0.10	0.08	≥ 0.05	1.03	0.84	1.25
Indebtedness^d^	No	0.47	0.09	25.73	<0.001	1.60	1.33	1.91

Notes: the reference category for the dependent variable is average level of physical activity. ^a^The reference category for steady income is NO. ^b^The reference category for per capita income is below USD 271. ^c^The reference category for savings is NO. ^d^The reference category for indebtedness is YES. *β*: assessment value of model parameters, SE: asymptotic standard error *β*, Wald *χ*^2^: parameter significance, p: Wald *χ*^2^ probability value, OR: odds ratio, and CI: confidence interval.

## Data Availability

The data used to support the findings of this study are available from the corresponding author upon request.

## Abbreviations

IPAQ-SF: International Physical Activity Questionnaire Short Form
S-ESQ: Socio-Economic Status of Working Age People Questionnaire
EEPA: Energy expenditure of physical activity
PAL: Physical activity level.
